# A Positive Feed Forward Loop between Wnt/*β*-Catenin and NOX4 Promotes Silicon Dioxide-Induced Epithelial-Mesenchymal Transition of Lung Epithelial Cells

**DOI:** 10.1155/2020/3404168

**Published:** 2020-12-08

**Authors:** Jia Ma, Qian Cai, Dandan Yang, Jiali Yang, Jing Xue, Miao Yu, Yingxue Liu, Fucheng Ma, Feng Li, Xiaoming Liu

**Affiliations:** ^1^Key Laboratory of Ministry of Education for Conservation and Utilization of Special Biological Resources in the Western, College of Life Science, Ningxia University, Yinchuan, Ningxia 750021, China; ^2^Key Laboratory of Environmental Factors and Chronic Disease Control, School of Public Health, Ningxia Medical University, Yinchuan 750004, China; ^3^General Hospital of Ningxia Medical University, Yinchuan, Ningxia 750004, China; ^4^Department of Anatomy and Cell Biology, University of Iowa, Iowa City, Iowa 52242, USA

## Abstract

Silicosis is a chronic fibrotic lung disease caused by the accumulation of silica dust in the distal lung. Canonical Wnt signaling and NADPH oxidase 4 (NOX4) have been demonstrated to play a crucial role in the pathogenesis of pulmonary fibrosis including silicosis. However, the underlying mechanisms of crosstalk between these two signalings are not fully understood. In the present study, we aimed to explore the interaction of Wnt/*β*-catenin and NOX4 of human epithelial cells in response to an exposure of silica dust. Results demonstrated an elevated expression of key components of Wnt/*β*-catenin signaling and NOX4 in the lungs of silicon dioxide- (SiO_2_-) induced silicosis mice. Furthermore, the activated Wnt/*β*-catenin and NOX4 signaling are accompanied by an inhibition of cell proliferation, an increase of ROS production and cell apoptosis, and an upregulation of profibrogenic factors in BEAS-2B human lung epithelial cells exposed to SiO_2_. A mechanistic study further demonstrated that the Wnt3a-mediated activation of canonical Wnt signaling could augment the SiO_2_-induced NOX4 expression and reactive oxygen species (ROS) production but reduced glutathione (GSH), while Wnt inhibitor DKK1 exhibited an opposite effect to Wnt3a. *Vice versa*, an overexpression of NOX4 further activated SiO_2_-induced Wnt/*β*-catenin signaling and NFE2-related factor 2 (Nrf2) antioxidant response along with a reduction of GSH, whereas the shRNA-mediated knockdown of NOX4 showed an opposite effect to NOX4 overexpression. These results imply a positive feed forward loop between Wnt/*β*-catenin and NOX4 signaling that may promote epithelial-mesenchymal transition (EMT) of lung epithelial cells in response to an exposure of silica dust, which may thus provide an insight into the profibrogenic role of Wnt/*β*-catenin and NOX4 crosstalk in lung epithelial cell injury and pathogenesis of silicosis.

## 1. Introduction

Silicosis is a fatal occupational chronic fibrotic lung disease caused by long-term exposure to respirable crystalline silica (silicon dioxide (SiO_2_)) dust that was ultimately deposited in distal airways [1, 2]. Due to the negligence and failure to control the risk of excessive silica exposure in modern industry and working activities using high-powered hand tools, such as denim sandblasting, jewellery polishing, artificial stone engineering, dental trimming, building constructing, and highway repairing, many developing countries including China are experiencing the reemergence of silicosis [3–5]. Unlike silicosis derived from the long-term silica exposure in traditional occupations such as mining, silica-related diseases in modern industries are characterized by an acute and accelerated progression owing to high-intensity silica dust concentrations and oxidative stress over a short time period [3].

Silicosis is currently an incurable disease, and its pathogenesis remains incompletely understood. Etiologically, the inhalation and deposition of silica dust induce inflammatory responses and the production of reactive oxygen species (ROS), which in turn lead to the epithelial-mesenchymal transition (EMT) and development of pulmonary fibrosis, which can be characterized by massive extracellular matrix (ECM) deposition and fibroblast proliferation, and myofibroblast differentiation [6]. Similar to that demonstrated in other chronic lung diseases, the pathogenesis of silicosis is controlled by interactions between various cellular signaling pathways [7–10]. Among them, the wingless-type MMTV-integration site (Wnt)/*β*-catenin signaling, a well-known critical cellular signaling pathway in embryonic development and tissue homeostasis, is reactivated in many chronic pulmonary diseases, including silicosis [8, 9, 11–13]. A blocking of Wnt/*β*-catenin signaling alleviated the lung inflammation and fibrosis in silica-induced mouse and rat silicosis models [14–16]. These studies suggested that Wnt/*β*-catenin is a key driver in the initiation and development of silicosis.

In addition to the dysregulation of cellular signaling activities, the inhalation of silica dust also causes oxidative stress by the production of reactive oxygen species (ROS) that contributes to chronic airway inflammation and epithelial cell injury [17]. Indeed, ROS are important mediators with a variety of biological functions, such as cell proliferation and differentiation, cell migration, and immune regulation [18]. ROS are also required for the maintenance and differentiation of primary lung fibroblasts for lung tissue homeostasis [19]. However, a continuously excessive production of ROS (oxidative stress) in the lung by nicotinamide adenine dinucleotide phosphate (NADPH) oxidase (NOX) may result in tissue injury and dysregulated injury/repair and ultimately lead to chronic pulmonary diseases, such as pulmonary fibrosis [19, 20]. In addition, exposure to silica dust was demonstrated to induce ROS production and lung injury in animal models [17, 21], suggesting a pathogenetic role of ROS in the development of silicosis.

The major ROS are generated by NOXs, membrane-bound enzyme complexes present in both phagocytes and nonphagocytic cells [22]. There are seven NOX homologs identified to date, namely, the NOX1-5 and dual oxidases 1 and 2 (DUOX1 and DUOX2) [18]. Despite the fact that these NOX proteins have abilities to produce superoxide anions, they possess distinct roles. Among them, NOX4 has a unique role and is broadly expressed in pulmonary artery endothelial cells and smooth muscle cells, airway epithelial cells, and pulmonary interstitial myofibroblasts. NOX4 is able to produce superoxide and generate extracellular H_2_O_2_ after a catalase activity [23, 24]. Moreover, NOX4 is most commonly implicated in profibrotic processes of multiple organs, including the liver and lung. In this regard, NOX4 is the only isoform of NOX proteins highly upregulated in the epithelial cells and myofibroblasts of lungs in idiopathic pulmonary fibrosis (IPF) patients [25, 26]. These clinical findings were corroborated by the fact that *NOX4*-deficient mice developed significantly less bleomycin-induced pulmonary fibrosis and alveolar epithelial cell death [25], suggesting the importance of NOX4 in the pathogenesis of pulmonary fibrosis. In addition, the inhibition of NOX4 attenuated pulmonary fibrosis in a bleomycin-induced rat lung fibrosis model [27]. Mechanistically, the excessive expression of NOX4 and production of ROS induce epithelial cell death, myofibroblast differentiation, and ECM deposition [23, 25].

In view of the widespread evidence for hyperactivated NOX4 and Wnt/*β*-catenin signaling in pulmonary fibrotic procession, and involvements of silica-induced ROS production in pathogenesis of silica-related diseases, we hypothesized that the interaction between the NOX4 and Wnt/*β*-catenin signaling may contribute to the pathogenetic process of silica-related lung disease. However, the link between Wnt/*β*-catenin signaling and NOX4-mediated ROS in the development of silicosis has not been established. Here, we demonstrate that a positive feed forward loop of NOX4 and Wnt/*β*-catenin signaling promotes the fibrotic property in airway epithelial cells in response to silica dust exposure.

## 2. Materials and Methods

### 2.1. Preparation of Silica (SiO_2_) Particles

Silica (SiO_2_, mesoporous, 2 *μ*m particle size, CAS number 7631-86-9) and silicon dioxide (~99% SiO_2_, 0.5-10 *μ*m particle size, 14808-60-7) were products of Sigma-Aldrich (St. Louis, MO, USA). The SiO_2_ particles were baked at 200°C for 2 h to inactivate endotoxin prior to being suspended in saline at a concentration of 100 mg/mL. The SiO_2_ saline stock was further dispersed for 15 min in a water bath sonicator followed by being triturated through a 25G needle before use as described in the previous report [28].

### 2.2. Generation of SiO_2_-Induced Silicosis Mouse Model

The protocol and use of mice were approved by the Laboratory Animal Committee of College of Life Science at Ningxia University, in accordance with guidelines of the National Institutes of Health *Guide for the Care and Use of Laboratory Animals* (NXULS20180123-3). For the generation of silicosis lungs in mice, the protocol, dose, and delivery method described in the previous study were employed with a slight modification [28]. Twelve healthy C57BL/6 mice with 6-8 weeks of age, male and female by half, were purchased from Beijing Vital River Laboratory Animal Technology Co. Ltd. (Beijing, China). All of the mice were housed in a Special Pathogen-Free (SPF) facility with a 12/12 h light/dark cycles and water *ad libitum* at Ningxia Medical University (Yinchuan, China). The male and female mice were randomly divided into two groups (3 male and 3 female mice per group): (1) saline control group: mice were intratracheally instilled 50 *μ*L of saline and (2) silica group: animals were intratracheally instilled 50 *μ*L of 50 mg/mL silica in saline (>99% SiO_2_, the dust particle size was 0.5–10 *μ*m, and 80% of the particles were 1–5 *μ*m). The mice were euthanized at 2 weeks (14 days) after the exposure of silica dust for pathohistological and molecular analysis [28].

### 2.3. Cell Cultures and Infection of Recombinant Adenovirus

A human bronchial epithelial cell line, BEAS-2B (ATCC CRL-9609), was purchased from American Type Culture Collection (ATCC) (Manassas, VA, USA). Cells were cultured in DME/F12 (50%/50% volume) basal medium (HyClone) supplemented with 10% Fetal Bovine Serum (FBS) (Ausbian, Cat No. VS500T, Australia) in a humidified atmosphere of 95% air-5% carbon dioxide (CO_2_) at 37°C. When cells reached ~80% confluence, they were utilized for infection with adenoviral vectors and/or exposed to different concentrations of SiO_2_ dust for various time periods for analysis. Adenoviral vectors expressing mouse Wnt3a (AdWnt3a), Ad.shRNA-NOX4 and adenoviral backbone vector control (AdC) were kindly provided by Dr. John F. Engelhardt at the University of Iowa (Iowa City, Iowa, USA) [29]. Adenoviral vector expressing human NOX4 (AdNOX4) was a product of Applied Biological Materials Inc. (Cat. No. 114456A; Richmond, Canada). Adenoviral vector AdDKK1, which expressed mouse DKK1, was generated by Shanghai Genechem Co., Ltd. (Shanghai, China). BEAS-2B cells were infected with adenoviral vectors at a multiplicity of infection of 1000 for 24 h before they were cultured for an additional 48 h in the presence or absence of SiO_2_ (2 *μ*m in size) at a concentration of 100 *μ*g/cm^2^. The cells were then harvested for analysis. For the treatment of ROS scavenger N-acetyl-L-cysteine (NAC), cells were refreshed with media containing 10 mmol/L of NAC for 2 h before SiO_2_ was added into the culture media. The cells were then continuously cultured in the presence of NAC for an additional 24 h or 48 h prior to being harvested for analysis.

### 2.4. Cell Viability Assay

The cell viability was accessed using the Cell Counting Kit- (CCK-) 8 as per the manufacturer's instruction (Dojindo Molecular Technologies, Kumamoto, Japan). Briefly, the cells (5 × 10^3^/well) were seeded in a 96-well plate and grown overnight prior to exposure to silica dust (2 *μ*m particle size) at a density of 0, 50, 100, 150, or 200 *μ*g/cm^2^ for 24 or 48 h. Subsequently, 10 *μ*L of CCK8 solution was added to each well, and the plates were incubated at 37°C for an additional 2 h. The absorbance of wavelength at 450 nm was read on a microplate reader (BioTek, Winooski, VT, USA). The relative cell viability was expressed as the percentage of (OD_SiO_2_−cells_ − OD_SiO_2_−medium_)/(OD_cells_ − OD_medium_) × 100, where the OD_SiO_2_−cells_, OD_SiO_2_−medium_, OD_cells_, and OD_medium_ represented the values of OD_450 nm_ of wells of SiO_2_-treated cells, medium containing SiO_2_, untreated control cell culture, and blank medium alone, respectively. All experiments were performed with biological triplicates, and data were representative of at least three independent experiments.

### 2.5. Western Blotting Analysis

The total protein of cells was extracted from cells treated with 100 *μ*g/cm^2^ of SiO_2_ for 48 h using cell lysis buffer (Kaiji Biotech Ltd., Beijing, China). The nuclear protein was extracted with a NE-PER™ Nuclear Extraction kit (Thermo Fisher Scientific China, Shanghai, China). The total protein of the mouse lung was isolated by homogenizing the tissue in Enhanced RIPA Lysis Buffer (Leagene Biotech Ltd., Beijing, China), followed by centrifugation. The cell lysate or homogenized lung tissue was then centrifuged at 12,000 × rpm for 20 min at 4°C; the supernatants were collected as total proteins. The concentration of protein was detected using the BCA Protein Assay Kit (Kaiji Biotech Ltd., Beijing, China). The proteins (30 *μ*g) were resolved by 8%-10% sodium dodecyl sulfate- (SDS-) polyacrylamide gel (SDS-PAGE) and transferred to a PVDF membrane (Millipore, Billerica, MA, USA). The membrane was then blocked in 5% nonfat milk in TBS for 1 hour at room temperature (RT). The protein of interest was probed with its specific antibodies, and the blots were then developed using the enhanced chemiluminescence (ECL) reagent (Amersham Biosciences, Piscataway, NJ, USA) as described elsewhere. The levels of protein expression were semiquantified by optical densitometry using ImageJ Software version 2.0.0 (http://rsb.info.nih.gov/ij/). The ratio between the net intensity of each sample divided by the GAPDH internal control was calculated as a densitometric arbitrary unit (A.U.), which served as an index of the relative expression of the protein of interest. The use and information of primary antibodies employed in the present study are listed in Suppl. Tables S1 and S2.

### 2.6. Histological and Immunofluorescent Staining

In order to histologically examine the pathology of the lungs of mice exposed to silica, the lung tissue of mice was fixed in 10% neutral formalin and processed paraffin embedding and section for pathohistological analysis by hematoxylin and eosin (HE) staining. Meanwhile, a part of the lung tissue was fixed in 4% paraformaldehyde (PFA) in PBS for 2 days prior to being embedded in an Optimal Cutting Temperature (OCT) compound for immunofluorescence (IF) staining on 10 *μ*m frozen tissue sections. For IF staining cells on coverslips, cells were seeded in the Glass Bottom Cell Culture Dish (diameter of 15 mm) at a density of 5 × 10^4^ cells/dish and cultured overnight prior to exposure to silica dust (100 *μ*g/cm^2^) for an additional 24 h or 48 h. Then, the cells were fixed with 4% PFA for 15 min. For IF staining, the 4% PFA-fixed slides were permeabilized by 0.2% TritonX-100/PBS for 20 min at RT, followed by being blocked with 5% normal donkey serum in PBS for 1 h at RT. The appropriately diluted primary antibody to the protein of interest was then applied to the section and incubated at 4°C overnight. The binding of primary antibody was detected by Alexa Flour fluorescence (488 or 565)-conjugated secondary antibodies. The EdU corporation assay was performed using the Click-iT™ EdU Cell Proliferation Kit as per the manufacturer's instruction (Cat# C10340, Thermo Fisher Scientific China, Shanghai, China). The slides were mounted with VectShield with DAPI medium (H-1200, Vector Laboratories, Burlingame, CA) for visualizing and imaging using a Leica TCS SP2 A0BS Confocal System and processed on Leica Confocal Software v.2.6.1 (Leica, Germany).

### 2.7. Flow Cytometry Analysis of ROS

Cells (1 × 10^5^ cells/well) cultured in a 6-well plate were infected with adenoviral vector for 24 h and/or treated with or without silica for an additional 48 h before the intercellular ROS was assessed by a flow cytometry assay. Briefly, the cells were dissociated from the plate and washed with 1x PBS, followed by being incubated with the CellROX® Orange Reagent (CellROX® Oxidative Stress Reagents, C10443, Invitrogen) at a final concentration of 5 *μ*M that was prediluted in phenol red-free DMEM in the dark at 37°C for 30 min. After the incubation, the CellROX® Orange Reagent solution was removed, and the removed medium and the cells were washed with PBS three times prior to being resuspended in PBS for flow cytometry analysis on a BD FACSCanto II. At least 10,000 events were analyzed for each condition. For ROS staining, cells were incubated with a 5 mmol/L CellROX® Orange Reagent in the dark at RT for 30 min, before they were imaged under fluorescence microscopy. All experiments were performed with biological triplicates, and data are representative of at least three independent experiments.

### 2.8. Measurement of Reduced Glutathione (GSH)

Cells were rinsed with PBS and lysed with 1% TritonX-100 in PBS for 30 min at 4°C. The lysis was collected and centrifuged at 3500 rpm for 10 min. The supernatant was harvested for analysis. A total of 100 *μ*L supernatant was employed to measure the value of OD_405 nm_ using the GSH Assay kit per the manual provided by the manufacturer (Jiancheng Institute of Biotechnology, Nanjing, China). The reduced GSH were normalized by protein concentrations. All experiments were performed with biological triplicates, and data are representative of at least three independent experiments.

### 2.9. Wnt/TCF Signaling Dual Luciferase Reporter Assay

The Wnt signaling TCF Reporter Plasmid (TopFlash) was a product of Millipore (Burlington, MA, USA), and the pCMV-renilla luciferase plasmid was purchased from Promega (Madison, WI, USA). In order to access the Wnt/*β*-catenin activity, BEAS-2B cells cultured in 12-well plates were cotransfected with TCF Reporter Plasmid (TopFlash) and pCMV-renilla luciferase plasmid (for internal control for normalization of transfection efficiency) at a ratio of 50 : 1 using X-tremeGENE HP (Roche, Penzburg, Germany). The cells were exposed to silica dust (2.0 nm size) at 24 h post the transfection and continued to culture for an additional 24 h or 48 h before they were harvested for analysis. The cells were lysed in 1x Passive Reporter Lysis Buffer (Promega). Protein concentrations were determined using the Bradford method, and all lysates were normalized to the same protein concentration using the lysis buffer. Two microliters of normalized cell lysate was used for measurement of the relative luciferase activity units (RLU), for both firefly and renilla luciferase, using the dual luciferase assay kit (Promega). Transfection efficiencies were normalized by dividing the relative firefly luciferase units by the relative renilla luciferase units. Following normalization, values were represented as RLU. All experiments were performed with biological triplicates, and data are representative of at least three independent experiments.

### 2.10. Statistical Analysis

All of the experiments were performed for at least three biological repeats. Data are presented as the mean ± standard error of the mean (SEM). All analyses were assessed using GraphPad Prism version 5 software (version 5.0, GraphPad Software Inc., La Jolla, CA, USA). Statistical significance was defined as *p* < 0.05.

## 3. Results

### 3.1. An Enhanced Wnt/*β*-Catenin Signaling Activity and Robust Expression NOX4 in Lungs of SiO_2_-Induced Silicosis Mice

**Figure 1:**
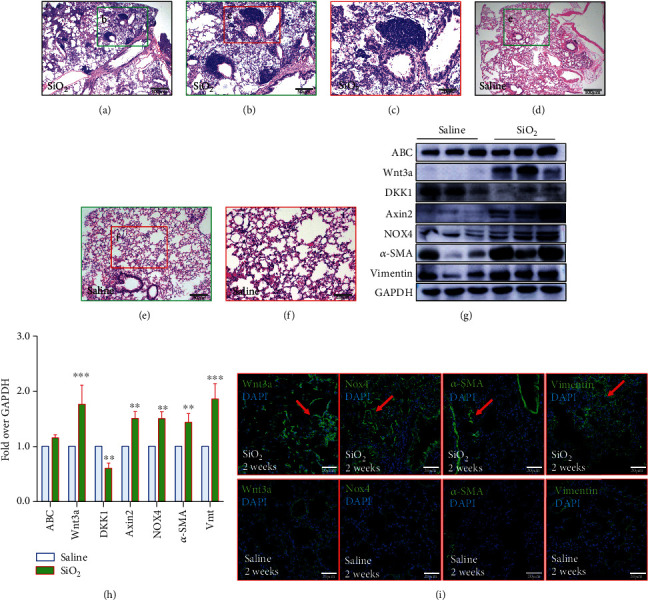
An elevated Wnt/*β*-catenin signaling activity and expression of NOX4 in lungs of mice exposed to silica (SiO_2_) dust. The lungs of C57BL/6 mice were harvested at 2 weeks post the exposure of saline control or silica dust (sizes ranged 0.5-10 *μ*m) for histological analysis by HE staining (a–f), examining alternations of the expression of signaling molecules by immunoblotting assay (g) and immunofluorescent staining (h). (a–f) Representative images of lungs of mice exposed to saline control (a–c) and silica dust (d–f). Abundant silicosis nodules were observed in lungs of mice challenged with silica dust (a–c), but not in the saline control group (d–f). (B, C, E, and F) Frame insets depict the regions of corresponding images shown in (b, c, e, f). (g) Immunoblotting analysis demonstrated an increased expression of NOX4 protein and an enhanced Wnt/*β*-catenin signaling activity as accessed by the increased abundance of active *β*-catenin (ABC) and Axin2 but a decreased Wnt inhibitor DKK1, accompanied by increased deposition of profibrogenic epithelial-mesenchymal transition (EMT) proteins alpha smooth muscle actin (*α*-SMA) and vimentin, in lungs exposed to silica. (h) Semiquantitative analysis of the expression of indicated proteins in (g) by evaluating the relative densitometric densities. (i) The increased expression of Wnt/*β*-catenin signaling ligand Wnt3a, NOX4, *α*-SMA, and vimentin was also predominantly detected in the silicosis nodules of silica-challenged lungs (top panel), compared to the saline (bottom panel) as ascertained by immunofluorescent staining (IF). DAPI was used for nuclear staining. Vmt: vimentin in (h). Bars in (a, d) 100 *μ*m; (b, e) 40 *μ*m; (c, f) 20 *μ*m; (i) 50 *μ*m. Data in (h) represented the mean ± SEM of 9 mice from 3 independent experiments. Compared to the control group, ^∗^*p* < 0.05, ^∗∗^*p* < 0.01, and ^∗∗∗^*p* < 0.001.

In order to understand the potential roles of Wnt/beta-catenin signaling and NOX4 in the development and progression of a silicotic lung, the expression of several key components of Wnt/*β*-catenin signaling cascade and NOX4 protein and the fibrotic and fibrogenic factors in the lungs of SiO_2_-induced silicosis mice were ascertained by immunoblotting (IB) and immunofluorescent staining (IF) assays. The mice that intratracheally received silica dust exhibited abundant silicosis nodules in the parenchyma of lungs as evaluated by HE histological staining (Figures 1(a)–1(c)). Such silicosis nodule was not observed in lungs of control mice challenged with saline (Figures 1(d)–1(f)). Of note, no difference in the pathogenesis of silicosis between male and female mice was observed. Molecular analysis using an immunoblotting assay uncovered that the NOX4 and Wnt/*β*-catenin signaling were elevated in silica-challenged lungs as accessed by an increased abundance of NOX4, active *β*-catenin (ABC), and Axin2 but decreased Wnt inhibitor DKK1 protein (Figures 1(g) and 1(h)). The enhanced activity of NOX4 and Wnt signaling was accompanied by an increased production of profibrogenic proteins alpha smooth muscle actin (*α*-SMA) and vimentin, in lungs exposed to silica (Figures 1(g) and 1(h)). Moreover, the increased abundances of Wnt3a, NOX4, *α*-SMA, and vimentin were further corroborated to be predominantly expressed in the silicosis nodules of silica-challenged lungs as determined by the immunofluorescent staining (IF) assay (Figure 1(i)). These results evidenced an involvement of NOX4 and Wnt/*β*-catenin signaling in the pathogenesis of the silicosis mouse lung.

### 3.2. SiO_2_ Activates Wnt/*β*-Catenin Signaling and Augments NOX4 in Lung Epithelial Cells

**Figure 2:**
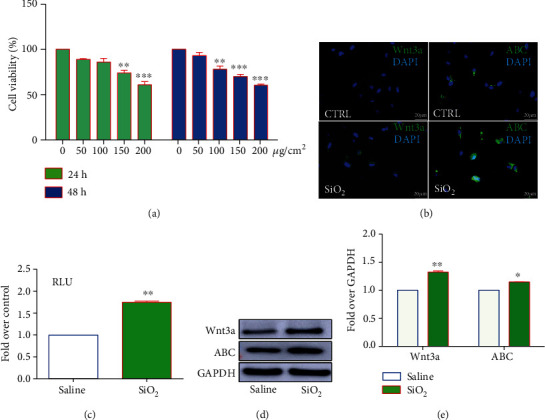
SiO_2_ activates Wnt/*β*-catenin signaling in lung epithelial BEAS-2B cells. The lung epithelial BEAS-2B cells were exposed to different doses of SiO_2_ (2 nm in size) for 24 h or 48 h, the cell viability (a) and Wnt/*β*-catenin signaling activity were examined. (a) A dose-dependent inhibition of cell viability was observed in BEAS-2B cells exposed to SiO_2_ for 24 h and 48 h as determined by a CCK8 assay. (b) Immunofluorescent staining further corroborated the increased expression of Wnt3a and active *β*-catenin (ABC) proteins in cells treated with 100 *μ*g/cm^2^ of SiO_2_ for 48 h in comparison with the saline control. (c) Dual luciferase reporter assay demonstrated a significantly enhanced SiO_2_-activated Wnt/*β*-catenin signaling in BEAS-2B cells exposed to 100 *μ*g/cm^2^ of SiO_2_ for 48 h compared with the saline control (CTRL) (*p* < 0.01). (d) Immunoblotting assay also revealed an increased abundance of Wnt3a and ABC in BEAS-2B cells exposed to 100 *μ*g/cm^2^ of SiO_2_ for 48 h relative to that of CTRL. (e) Semiquantitative analysis of the expression of indicated proteins in (d) by evaluating the relative densitometric densities. Bars in (b) 20 *μ*m. Compared to the CTRL, ^∗^*p* < 0.05 and ^∗∗^*p* < 0.01.

Since the epithelial-mesenchymal transition (EMT) or myofibrogenesis of lung epithelial cells is a hallmark of pulmonary fibrosis, a feature of silicosis [30], the alteration of Wnt/*β*-catenin signaling activity and NOX4 expression of BEAS-2B lung epithelial cells in response to silica dust was examined. The cell viability assay suggested a 50% decrease of cell viability in lung epithelial BEAS-2B cells exposed to 200 *μ*g/cm^2^ of SiO_2_ and revealed a dose-dependent inhibition of cell proliferation in cells exposed to SiO_2_ for 24 h and 48 h in a range of 0-200 *μ*g/cm^2^ (Figure 2(a)). As expected, the exposure of SiO_2_ (100 *μ*g/cm^2^) led to a significantly enhanced activation of Wnt/*β*-catenin signaling in BEAS-2B cells compared to the saline control (*p* < 0.01), as assessed by IF staining of Wnt3a ligand in the cytoplasm and ABC in nuclei (Figure 2(b)), the Wnt/Tcf-Lef transcriptional activity using a dual luciferase reporter assay, a readout of Wnt/*β*-catenin signaling (Figure 2(c)), and immunoblotting (IB) assay (Figures 2(d) and 2(e)). The elevated Wnt activity was accompanied by an increased abundance of Wnt/*β*-catenin signaling ligand Wnt3a and mediator nuclear active *β*-catenin (ABC), which was corroborated by the IF staining (Figure 2(b)) and IB assay (Figures 2(d) and 2(e)).

**Figure 3:**
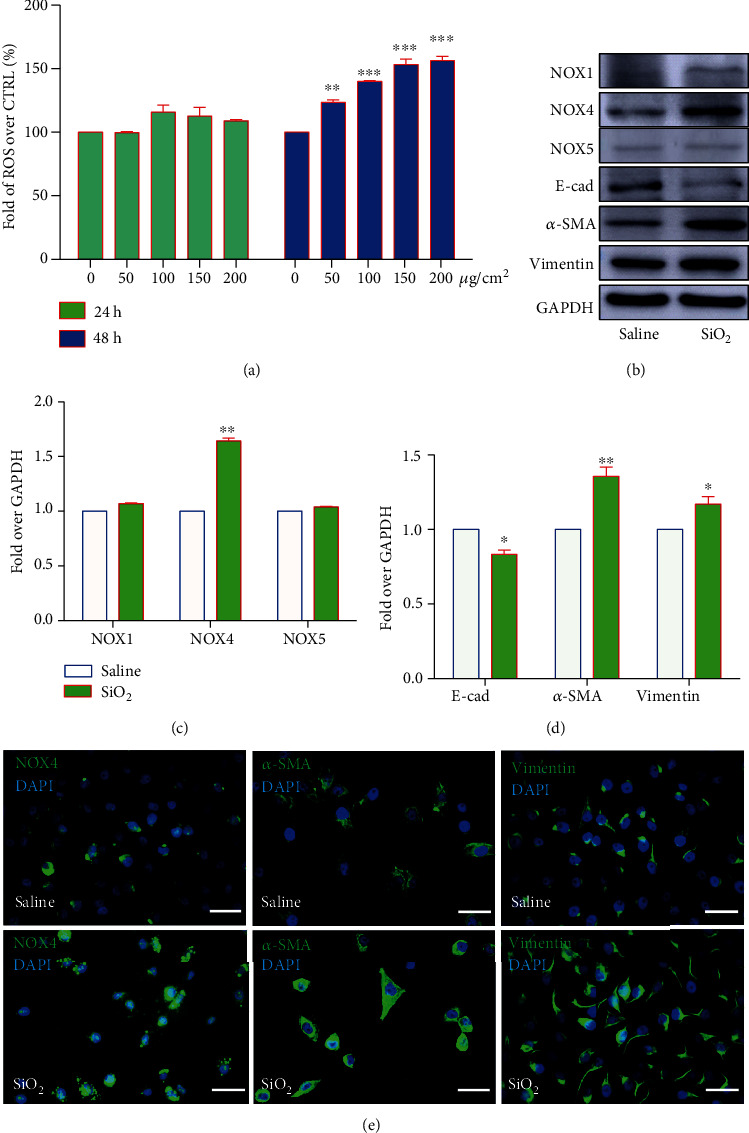
SiO_2_ alters the NOX-mediated ROS production and promotes EMT and cell apoptosis in lung epithelial BEAS-2B cells. The lung epithelial BEAS-2B cells were exposed to different doses of SiO_2_ (2 nm in size) for 24 h or 48 h; the alteration of ROS production (a) and expression of NOX and EMT proteins were examined. (a) A dose- and time-dependent increase of ROS production induced by SiO_2_ was determined in BEAS-2B cells at 48 h but not 24 h post exposure to silica dust. (b) Immunoblotting assay revealed an increased expression of NOX4 and *α*-SMA but reduced expression of E-cadherin in BEAS-2B cells exposed to 100 *μ*g/cm^2^ of SiO_2_ for 48 h relative to the saline. (c) Semiquantitative analysis of the expression of NOX1, NOX4, and NOX5 proteins in (b) by evaluating the relative densitometric densities. (d) Semiquantitative analysis of the expression of EMT-related proteins E-cadherin, *α*-SMA, and vimentin in (b) by evaluating the relative densitometric densities. (e) Representative images of IF exhibited more abundant NOX4, *α*-SMA, and vimentin proteins in BEAS-2B cells exposed to 100 *μ*g/cm^2^ of SiO_2_ for 48 h as compared with the saline control. Bars in (c) 20 *μ*m. Compared to the CTRL, ^∗∗^*p* < 0.01; ^∗∗∗^*p* < 0.001.

In order to examine the potential of SiO_2_ in the induction of ROS production and EMT of epithelial cells, we examined the expression of NOX proteins and EMT-related molecules in cells exposed to silica for both 24 h and 48 h, although a significant reduction of cell viability was induced by the exposure of SiO_2_ at 100 *μ*g/cm^2^ for 48 h. As expected, a dose-dependent increase of ROS production was observed in BEAS-2B cells treated with SiO_2_ for 48 h, but not 24 h post the exposure of silica dust relative to the saline (Figure 3(a)), suggesting inhibition of cell proliferation in cells exposed to SiO_2_. As NOX proteins were the major source of intracellular ROS [31], alterations of several NOX family proteins were examined in BEAS-2B cells upon SiO_2_ stimulation. Indeed, the increase of ROS production was along with an increased abundance of NOX4 and profibrogenic factors *α*-SMA and a reduction of E-cadherin protein (Figures 3(b)–3(d)). Of note, SiO_2_ failed to induce the expression of NOX1 and NOX5 as determined by the IB assay (Figures 3(b) and 3(c)). The SiO_2_-induced expression of NOX4 and *α*-SMA and another profibrogenic protein vimentin was further validated by IF in BEAS-2B cells (Figure 3(e)).

**Figure 4:**
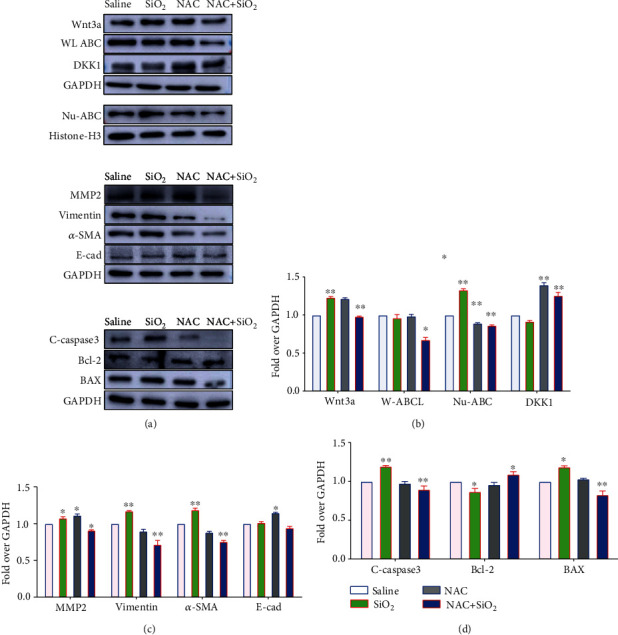
ROS scavenger NAC inhibits SiO_2_-induced Wnt/*β*-catenin signaling activity and expression of fibrogenic factors. The lung epithelial BEAS-2B cells were exposed to 100 *μ*g/cm^2^ of SiO_2_ (2 *μ*m in size) for 48 h in the presence or absence of ROS scavenger NAC; the abundance of the indicated Wnt/*β*-catenin signaling key molecules, fibrogenic factors, and signaling of caspase-3-mediated cell apoptosis were examined by an immunoblotting assay. (a) Representative immunoblots showed that the presence of NAC reduced the SiO_2_-induced Wnt/*β*-catenin signaling ligand Wnt3a and mediator nuclear ABC but increased the expression of Wnt inhibitor DKK1 (top panel); suppressed the SiO_2_-induced fibrogenic factors MMP2, vimentin, and *α*-SMA but increased the abundance of epithelial cell marker E-cadherin (middle panel); and decreased the SiO_2_-induced abundance of cleaved caspase-3 and BAX but increased the expression of antiapoptotic protein Bcl-2 in BEAS-2B cells exposed to 100 *μ*g/cm^2^ of SiO_2_ for 24 h relative to the saline control (bottom panel). (b) Semiquantitative analysis of the expression of Wnt signaling proteins Wnt3a, ABC, and DKK1 in (a) by evaluating the relative densitometric densities using arbitrary units (A.U.). (c) Semiquantitative analysis of the expression of fibrogenic factors MMP2, vimentin, *α*-SMA, and E-cadherin in (a) by evaluating the relative densitometric densities using arbitrary units (A.U.). (d) Semiquantitative analysis of the expression of apoptotic marker cleaved caspase-3 and BAX and antiapoptotic protein Bcl-2 in (a) by evaluating the relative densitometric densities using arbitrary units (A.U.). Data were expressed as the mean ± SEM from three independent experiments. Compared to the CTRL, ^∗∗^*p* < 0.01; ^∗∗∗^*p* < 0.001. WL-ABC: whole cell lysate active beta-catenin; Nu-ABC: nuclear active beta-catenin.

Intriguingly, the ROS scavenger N-acetyl-L-cysteine (NAC) (10 mmol/L) exhibited a capacity to diminish SiO_2_-activated Wnt/*β*-catenin signaling as determined by the altered abundance of nuclear ABC protein, although no significant change of nuclear ABC was detected in cells treated with NAC alone (Figure 4(a) ). Notably, the NAC treatment also displayed the ability to reduce SiO_2_-induced EMT and fibrogenic proteins MMP2, *α*-SMA, and vimentin and EMT suppressor E-cadherin, despite the fact that SiO_2_ or NAC alone failed to significantly alter the expression of these proteins in BEAS-2B cells (Figure 4). Of note, in addition to the inhibition of cell proliferation, the exposure of SiO_2_ also induced cell apoptosis as determined by an increased expression of caspase-3-mediated proapoptotic proteins including cleaved caspase-3 and BAX, but a reduced expression of antiapoptotic protein relative to the saline control (Figures 4(a) and 4(d)). The presence of NAC could reduce the expression of SiO_2_-induced apoptotic proteins (Figures 4(a) and 4(d)), suggesting that the exposure of silica could inhibit cell proliferation and induce cell apoptosis in BEAS-2B cells. Together, these data suggested that the SiO_2_-induced NOX4 and ROS generation played a pivotal role in the EMT and fibrogenesis in lung epithelial cells during silicosis, which also implied an underlying mechanism by which interaction between the Wnt/*β*-catenin signaling and NOX4 of lung epithelial cells was implicated in the pathogenesis of silicosis lungs.

### 3.3. Wnt/*β*-Catenin Signaling Alters NOX4-Mediated ROS Production in Lung Epithelial Cells

**Figure 5:**
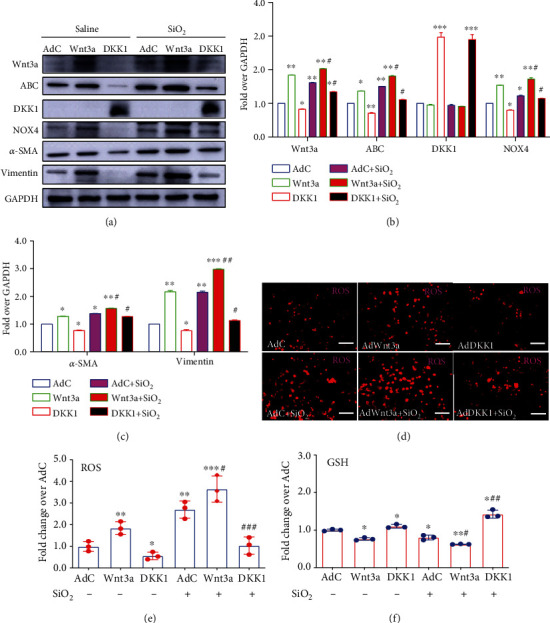
Impacts of Wnt/*β*-catenin signaling on the expression of NOX4 and ROS production in lung epithelial cells in response to SiO_2_ exposure. The lung epithelial BEAS-2B cells were infected with adenoviral vectors at multiplicity of infection of 1000 for 24 h before they were exposed to SiO_2_ and cultured for additional 48 h, the abundance of NOX4 and fibrogenic factors were examined by an immunoblotting assay (a–c), and production of ROS (d, e) and GSH (f) was determined by FACS and/or fluorescent staining. (a) Representative images of immunoblots of indicated proteins of interest showed the, respectively, increased and decreased expression of NOX4 and fibrogenic factors in cells infected with AdWnt3a and AdDKK1 regardless of SiO_2_ exposure, in comparison with cells infected by AdC control virus (CTRL). (b) Semiquantitative analysis of the expression of Wnt signaling proteins Wnt3a, ABC, DKK1, and NOX4 in (a) by evaluating the relative densitometric densities using arbitrary units (A.U.). (c) Semiquantitative analysis of the expression of fibrogenic factors *α*-SMA and vimentin in (a) by evaluating the relative densitometric densities using arbitrary units (A.U.). (d) Representative images of cells treated with indicated conditions and stained with 5 mmol/L CellROX® Orange Reagent images displayed more robust ROS staining in cells exposed to SiO_2_ compared to cells without SiO_2_ exposure; a, respectively, increased and decreased intensive ROS staining was observed in AdWnt3a and AdDKK1-infected cells relative to AdC-infected cells. (e) Quantitative analysis showed that the Wnt3a-mediated activation of Wnt/*β*-catenin signaling increased the ROS production in lung epithelial cells, regardless of the exposure to SiO_2_, and inhibition of the Wnt signaling by AdDKK1 infection dramatically reduced the ROS production, including the SiO_2_-induced ROS in these cells, as compared with the AdC-infected cell controls. (f) Effects of Wnt/*β*-catenin signaling in the production of reduced glutathione (GSH) in lung epithelial cells. In addition to the reduction of GSH by SiO_2_, an activation of Wnt/*β*-catenin signaling by Wnt3a further reduced the production of GSH, and the DKK1-mediated inhibition of Wnt signaling increased the GSH production in lung epithelial cells, regardless of the exposure to SiO_2_, as compared with the AdC-infected cells. Data are presented as the mean ± SEM of at least three repeat experiments. ^∗^*p* < 0.05, ^∗∗^*p* < 0.01, and ^∗∗∗^*p* < 0.001 compared with AdC-infected cells without SiO_2_ exposure; ^#^*p* < 0.05, ^##^*p* < 0.01, and ^###^*p* < 0.001 compared to AdC-infected cells with SiO_2_ exposure. Bars in (d) 50 *μ*m.

In order to investigate the interaction between the Wnt/*β*-catenin signaling and NOX4 in lung epithelial cells, the signaling activity was altered in BEAS-2B cells by the infection of adenoviral vector expressing mouse Wnt3a (AdWnt3a) or DKK1 (AdDKK1), and the change of ROS production in response to SiO_2_ exposure was measured. The IB assay showed a robust expression of Wnt3a and DKK1 proteins in cells infected with AdWnt3a and AdDKK1, along with an increase and decrease of ABC protein in comparison to cells infected by control AdC vector, respectively (Figures 5(a) and 5(b)). Notably, the AdWnt3a-mediated activation of Wnt/*β*-catenin signaling resulted in an increased *α*-SMA and vimentin proteins, but a reduced E-cadherin (Figures 5(a) and 5(c)). Of note, the Wnt3a or DKK1 alone was able to alter the NOX4 expression and ROS productions in BEAS-2B cells, regardless of the exposure of SiO_2_ (Figures 5(a)–5(e) ), despite the fact that SiO_2_ alone also significantly induced ROS generation, as demonstrated by both the fluorescent staining with CellROX® Orange Reagent (Figure 5(d)) and FACS quantitative assay (Figure 5(e)). In addition, the activated Wnt signaling also inhibited the production of reduced glutathione (GSH) compared to AdC-infected cells (Figure 5(f)). As expected, the AdDKK1-mediated inhibition of Wnt/*β*-catenin signaling led to an opposite effect to Wnt3a (Figure 5). These results suggested a mechanism by which the SiO_2_-activated Wnt/*β*-catenin signaling induced NOX4 expression, which substantially increased the ROS production and the expression of EMT and fibrogenic proteins in lung epithelial cells.

### 3.4. NOX4 Enhances Wnt/*β*-Catenin Signaling and Promotes EMT in Lung Epithelial Cells

**Figure 6:**
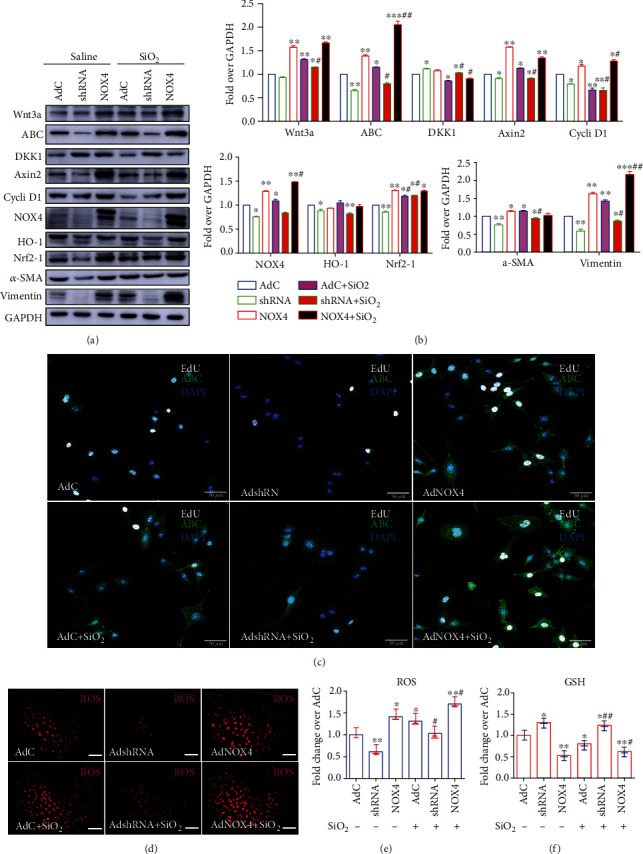
Effects of NOX4 on Wnt/*β*-catenin signaling and EMT of lung epithelial cells in response to SiO_2_ exposure. The lung epithelial BEAS-2B cells were infected with indicated adenoviral vectors at multiplicity of infection of 1000 for 24 h before they were exposed to SiO_2_ and cultured for additional 48 h; the expression of Wnt/*β*-catenin signaling key molecules and fibrogenic factors (a, b) and the production of GSH (c) were examined. (a) Representative images of immunoblots of indicated proteins of interest. An overexpression of NOX4 in BEAS-2B cells resulted in an increased abundance of Wnt/*β*-catenin signaling proteins Wnt3a, ABC, cyclin D1, and Axin2, but less abundant Wnt inhibitor DKK1, along with an increased expression of fibrogenic factors, and oxidative stress-related proteins HO-1 and Nrf-2. A shRNA-mediated knockdown of NOX4 led to an opposite effect of the overexpression of NOX4. (b) Semiquantitative analysis of the expression of proteins of Wnt signaling cassette (top panel), NOX4, HO-1, and Nrf-2 (bottom left panel) and fibrogenic factors (bottom right panel) in (a) by evaluating the relative densitometric densities using arbitrary units (A.U.). (c) Representative images of IF exhibited an activation of Wnt signaling mediated by NOX4 in BEAS-2B cells regardless of the exposure of SiO_2_ as ascertained by the expression of ABC and cell proliferation by EdU incorporation, while shRNA-mediated knockdown of NOX4 acted an opposite effect, as compared with the AdC control. (d) Representative images of ROS staining showed more robust ROS staining in cells infected with AdNOX4, while shRNA-mediated knockdown of NOX4 exhibited an opposite effect to AdNOX4, as compared with the saline and AdC controls. (e) Quantitative analysis showed the NOX4 further SiO_2_-induced increased the ROS production in lung epithelial cells infected with AdNOX4, while shRNA-mediated knockdown of NOX4 exhibited an opposite effect to AdNOX4, as compared with the saline and AdC controls. (f) Impacts of NOX4 in the production of reduced glutathione (GSH) in lung epithelial cells. The overexpression of NOX4 further reduced the production of GSH, and the shRNA-mediated suppression of NOX4 increased the GSH production in lung epithelial cells, as compared with the saline treatment and AdC-infected cells. Data are presented as the mean ± SEM of at least three repeat experiments. ^∗^*p* < 0.05, ^∗∗^*p* < 0.01, and ^∗∗∗^*p* < 0.001 compared with AdC-infected cells without SiO_2_ exposure; ^#^*p* < 0.05, ^##^*p* < 0.01, and ^###^*p* < 0.001 compared to AdC-infected cells with SiO_2_ exposure.

Next, we sought to explore whether NOX4 was able to alter Wnt/*β*-catenin signaling in lung epithelial cells in response to silica dust. To this end, the function of NOX4 in BEAS-2B cells was altered by infections of adenoviral vectors expressing NOX4 (AdNOX4) or short hairpin RNA (shRNA) to *NOX4* gene (AdshRNA). As expected, an increased abundance of NOX4 protein was found in cells infected with AdNOX4, and a decreased NOX4 protein was detected in cells infected with AdshRNA, suggesting that these adenoviral vectors were capable of overexpressing and knocking down NOX4 in BEAS-2B cells (Figures 6(a) and 6(b)). Consequentially, the AdNOX4-mediated overexpression of NOX4 activated Wnt/*β*-catenin signaling as determined by an increased abundance of Wnt3a, ABC, cyclin D1, and Axin2. The activation of Wnt/*β*-catenin signaling was further corroborated by IF of ABC (Figure 6(c)), and the expression of NOX4 increased the EdU incorporation in BEAS-2 cells, implying that NOX4 was able to promote epithelial cell proliferation (Figure 6(c)). Of interest, the overexpression of NOX4 significantly inhibited DKK1 protein in BEAS-2 cells (Figures 6(a) and 6(b)). As seen in the above AdWnt3a-infected cells, the NOX4-activated Wnt/*β*-catenin signaling also increased the expression of fibrogenic factors, and oxidative stress-related proteins NFE2-related factor 2 (Nrf2) in BEAS-2B cells, but the expression of Nfr2-regulated gene heme oxygenase-1 (HO-1) has not altered the ectopic expression of NOX4 (Figures 6(a) and 6(b)). In contrast to that seen in cells overexpressing NOX4, the expression of the above-examined proteins in cells with shRNA-mediated NOX4 knockdown was opposite (Figures 6(a)–6(c)). Consistent with the Wnt3a-mediated Wnt activation, the overexpression of NOX4 increased the ROS production (Figures 6(d) and 6(e)), while suppressing the production of reduced glutathione (GSH) (Figure 6(f)), and a knockdown of NOX4 reduced ROS production but induced GSH production in BEAS-2B cells regardless of SiO_2_ stimulation (Figures 6(d)–6(f)). These data suggested that the SiO_2_-induced NOX4 expression could enhance Wnt/*β*-catenin signaling activity, which in turn increased ROS generation and reduced GSH, which ultimately induced cell injury and promoted the EMT in lung epithelial cells.

## 4. Discussion

Silicosis is characterized as a chronic fibrotic lung disease caused by repeated inhalation of excessive silica dust. The exposure of silica insults induces sustained inflammations and oxidative stress, resulting in a direct or indirect injury to the alveolar epithelium in distal lungs. In response to the injury, the epithelial cells are able to repair by initiating the injury/repair processes that are tightly regulated by interactions between varied cellular signaling pathways. However, dysregulation of these signaling may lead to the initiation of fibrotic response in pulmonary fibrosis [32]. Among these signaling, the Wnt signaling was linked with the development of fibrosis [9] and was altered by silica dust in lung epithelial cells *in vitro* and silicosis animal model *in vivo*, through mechanisms by which it interacted with other signaling or molecules and contributed to profibrogenic and inflammatory responses in lung epithelial cells [13–15, 33].

In the present study, the interaction between the Wnt/*β*-catenin signaling and NOX4 in the proliferation and profibrogenic response to SiO_2_ in human epithelial cells was interrogated. The results demonstrated that both Wnt/*β*-catenin and NOX4 signaling were elevated in the lungs of SiO_2_-induced silicosis mice. The exposure of SiO_2_ led to an inhibition of cell proliferation and induction of cell apoptosis, activation of Wnt/*β*-catenin signaling, induction of the NOX4 and ROS production, and the expression of EMT-related proteins in BEAS-2B human lung epithelial cells. Molecular analysis further revealed that the Wnt3a-mediated activation of Wnt/*β*-catenin further increased the SiO_2_-induced NOX4 expression and ROS production but reduced GSH, while Wnt inhibitor DKK1 exhibited an opposite effect to Wnt3a. *Vice versa*, an ectopic expression of NOX4 enhanced the activity of Wnt/*β*-catenin signaling and reduction of GSH, whereas the shRNA mediated knockdown of NOX4-enervated Wnt signaling activity and increase of GSH. Mechanistically, the NFE2-related factor 2 (Nrf2) antioxidant response was involved in the crosstalk between the Wnt/*β*-catenin and NOX4 signaling in BEAS-2B cells in response to SiO_2_ challenge.

Wnt signaling is pivotal to lung development and homeostatic maintenance of the mature lung, by mediating stem cell self-renewal, turnover, and injury/repair of epithelia [34, 35]. However, accumulating evidences have shown that Wnt has been implicated in many types of pulmonary diseases, and a dysregulated Wnt signaling in mature lungs was recognized as a driver that leads to excessive cell proliferation and improper cell differentiation for fibrotic repair [36, 37]. Indeed, a reactivated Wnt was identified as a key contributor in the initiation and development of hyperproliferative chronic pulmonary diseases [9, 12], such as idiopathic pulmonary fibrosis (IPF) [11, 38], asthma [39], and COPD [8, 11, 34, 40]. With respect to silicosis, both *β*-catenin-mediated canonical signaling and *β*-catenin-independent noncanonical signaling were altered in human airway epithelial cells upon silica stimulation; the Wnt inhibitor SFRP1 and noncanonical ligand Wnt5a were downregulated, while another Wnt inhibitor DKK1 was upregulated [13], despite the fact that the canonical Wnt/*β*-catenin signaling was reactivated in silicosis lungs [14, 15]. Therefore, inhibition of Wnt/*β*-catenin signaling by shRNA to *β*-catenin [14, 15] displayed a capability to dramatically alleviate silica-induced fibrosis in a silicosis mouse model [16]. Such attenuation of silica-induced pulmonary fibrosis by targeting Wnt/*β*-catenin signaling was also reported in silicosis rats that were administrated with rat bone marrow mesenchymal stromal cells (BMSCs) [16]. In line with the above findings, an enhanced Wnt/*β*-catenin signaling, along with the increased abundance of NOX4 and profibrogenic proteins but reduced DKK1, was also observed in a SiO_2_-induced silicosis mouse lung, and BEAS-2B human lung epithelial cells exposed to SiO_2_.

Repeated oxidative stress is one of the detrimental factors of lung injury. The NOX-mediated overproduction of ROS due to excessive stimulation of proinflammatory cytokines or environmental insults such as silica dusts causes a major part of oxidative stress in the lungs. Among different isoforms of NOXs, the pathophysiological roles of NOX4 isoform have a great implication in lung epithelial cell death, (myo)fibroblast differentiation, and collagen deposition [23, 25, 41]. In this regard, an elevated NOX4 was observed in hyperplastic alveolar type II cells and contributed to the cell death and robustly expressed in pulmonary fibroblasts of IPF patients and epithelial cells [25, 26]. Experimentally, mice with deficient *NOX4* exhibited a significantly less severe fibrotic phenotype in the lungs of a bleomycin-induced pulmonary fibrosis mouse model [25], suggesting that NOX4 was a potential target for treatment of pulmonary fibrosis including silicosis. Indeed, in a previous study examining the effect of Tanshinone IIA (Tan IIA), a natural compound of traditional Chinese medicine in a silicosis rat model, Feng et al. found that Tan IIA could significantly alleviate the silica-induced pulmonary fibrosis, by reducing the silica-augmented NOX4 and enhancing Nrf2/ARE antioxidant activity in the lung of silicosis rats [42]. In this report, an increased expression of NOX4 and ROS production was also observed in BEAS-2B lung epithelial cells in response to SiO_2_ stimulations. Of note, the shRNA-mediated knockdown of NOX4 led to a significant decrease of SiO_2_-induced profibrogenic molecules (*α*-SMA, vimentin) in lung epithelial cells.

An increasing number of evidences have demonstrated a crosstalk of Wnt/*β*-catenin and ROS in the regulation of cell proliferation and differentiation [43–46]. For example, a previous study has shown that Wnt/*β*-catenin was able to modulate redox regulatory protein p66(Shc), which in turn regulate a NOX4-mediated ROS and ultimately lead to vascular endothelial dysfunction [43]. In this context, the Wnt3a-induced Wnt signaling, NOX4 expression, and ROS production could be inhibited by p66(Shc) knockdown and antioxidant NAC, whereas an overexpression of p66(Shc) enhanced Wnt signaling. A constitutive activation of Wnt/*β*-catenin in the endothelium resulted in an increased vascular ROS production and endothelial dysfunction [43]. In contrast, ROS also show a capacity in modulating Wnt/*β*-catenin signaling in mouse extraembryonic endoderm patterning; a sustained exposure of H_2_O_2_ (ROS) enhanced Wnt/*β*-catenin activity in retinoic acid-treated F9 teratocarcinoma cells and promoted cell differentiation into primitive endoderm, while exposure of these cells to antioxidant NAC impeded cell differentiation [44]. Similarly, the cell oxidative injury induced by a reactivation of Wnt/*β*-catenin signaling was also observed in podocyte dysfunction, and a podocyte-specific knockout of *β*-catenin protected podocytes from injury and albuminuria induced by advanced oxidation protein products in mice. Mechanistically, the Wnt/*β*-catenin exerted its function by inducing the receptor of advanced glycation end products- (RAGE-) mediated NOX expression, ROS production, and activation of nuclear factor-kappaB (NF*κ*B) [45]. Such a NOX-mediated regulation of Wnt/*β*-catenin was also reported in intestinal and colon epithelial cells, in which the NOX1 regulated Wnt signaling in a redox-dependent manner [46]. In consonance with the above findings, in the present study, the crosstalk of Wnt/*β*-catenin and NOX4 was also found in BEAS-2B lung epithelial cells in response to silica dust exposure. The Wnt3a-activated Wnt/*β*-catenin signaling augmented NOX4 expression and ROS production and reduced GSH production, accompanied by an increased expression of profibrogenic proteins; conversely, the DKK1-mediated inhibition of Wnt/*β*-catenin exerted an opposite effect of Wnt3a on lung epithelial cells exposed to SiO_2_. *Vice versa*, an overexpression of NOX4 enhanced Wnt/*β*-catenin signaling activity, along with an augmentation of profibrogenic proteins in cells. NOX4 also reduced GSH production and increased the expression of Nrf2, a key intracellular antifibrotic factor that maintains the homeostasis of ECM [47], while the shRNA-mediated knockdown of NOX4 demonstrated an opposite effect of NOX4 overexpression. This result was in agreement with the finding in cystic fibrosis transmembrane conductance regulator (CFTR) defected cells with ROS overproduction [48]. It is worthwhile to note that the overexpression of NOX4 suppressed the DKK1 expression. This observation was in line with the result of the reduction of DKK1 in the lung of silicosis mice (Figure 1(b)), implying that the overwhelming silica-induced NOX4 in the silicotic lungs might inhibit the DKK1 expression. This result thus suggests that DKK1 may be a novel target in silicosis, which requires further investigation.

In addition, reduced glutathione (GSH) is a ubiquitous tripeptide thiol, which has been recognized as a protective antioxidant against oxidative stresses [49]. A deficiency of GSH in the lower respiratory tract was thought to have an important implication in the progression of IPF [50]; it was downregulated in bleomycin-induced lung fibrosis in a mouse [51] and PM2.5-induced pulmonary fibrosis lung in rats [52]. In this study, a Wnt/*β*-catenin-mediated decline of reduced glutathione (GSH) was also determined in lung epithelial cells, which was in line with findings in lens epithelial cells [53], zebrafish ZF4 cells [54], and murine macrophage-like RWA264.7 cells [55]. Taken together, these data suggest that a positive forward loop between Wnt/*β*-catenin and NOX4 promotes the EMT and fibrogenesis in lung epithelial cells in response to the exposure of silica dust.

## 5. Conclusion

**Figure 7:**
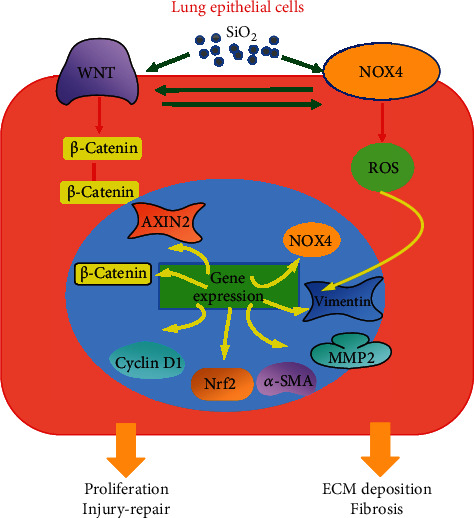
A schematic mechanism of interaction between Wnt/*β*-catenin and NOX4 signaling in SiO_2_-induced epithelial-mesenchymal transition in lung epithelial cells. The exposure of silica dust activated Wnt/*β*-catenin signaling and induced NOX4 expression in lung epithelial cells, substantially promoted the expression of Wnt target genes for epithelial cell proliferation and injury repair, and increased ROS production and expression of extracellular matrix (ECM) and fibrogenic factors to promote fibrogenesis. However, the activation of Wnt/*β*-catenin signaling also induced NOX4 expression that resulted in an overwhelming ROS production and further caused epithelial cell injury. *Vice versa*, the NOX4 (or ROS) was able to in turn enhanced Wnt/*β*-catenin signaling which further induced NOX4 expression. In this context, the Wnt/*β*-catenin signaling and NOX4 formed a positive forward loop to promote lung epithelial injury and EMT, ECM deposition, and fibrogenesis in response to a continuous exposure of silica dust.

Collectively, in the present study, the crosstalk between canonical Wnt signaling and NOX4 was investigated in BEAS-2B human lung epithelial cells in response to SiO_2_ particles. The *in vivo* results demonstrated an enhanced activity of Wnt/*β*-catenin and NOX4 signaling in SiO_2_-induced mouse silicosis lungs. The *in vitro* study revealed the inhibition of cell proliferation, along with enhanced Wnt/*β*-catenin signaling activity, increased ROS production, expression of NOX4, and profibrogenic proteins in BEAS-2B cells exposed to SiO_2_. A molecular study further demonstrated the crosstalk between canonical Wnt/*β*-catenin signaling and NOX4 in lung epithelial cells exposed to silica dust; an activation of Wnt/*β*-catenin signaling increased NOX4 and ROS production but reduced GSH; *vice versa*, an increased NOX4 enhanced the canonical Wnt signaling. Mechanistically, the Nrf2-mediated antioxidant activity may be involved in the interaction between the Wnt/*β*-catenin and NOX4 signaling and play roles in the EMT and fibrogenic processes of lung epithelial cells in response to a silica exposure (Figure 7). These findings imply that a positive feed forward loop between Wnt/*β*-catenin and NOX4 signaling promotes EMT property in BEAS-2B cells exposed to silica stimulation, which thus emphasize the profibrogenic role of the crosstalk of Wnt and NOX-mediated ROS in lung epithelial cell injury and development of silicosis, which warrants further investigations.

## Data Availability

All data generated or analyzed during this study are included in this published article.

## Abbreviations

ALI: Acute lung injury carcinoma
ARDS: Acute respiratory distress syndrome
ARE: Antioxidant response element
BAX: Bcl-2-associated X
Bcl-2: B cell lymphoma-2
BM: Bone marrow
BMSCs: Bone marrow mesenchymal stem cells
CM: Conditioned medium
ECL: Enhanced chemiluminescence
EVs: Extracellular vesicles
hfPMSCs: Human fetal placental mesenchymal stem cells
HGF: Hepatocyte growth factor
HO-1: Glutathione peroxidase
keap1: Kelch-like ECH-associated protein 1
Nrf2: Nuclear factor erythroid 2-related factor 2
MSCs: Mesenchymal stem cells
PVECs: Pulmonary microvascular endothelial cells
ROS: Reactive oxygen species
SLE: Systemic lupus erythematosus
T-AOC: Total antioxidative capacity.

## Supplementary Materials

Supplementary Materials Suppl. Table S1: primary and secondary antibodies used for immunostaining. Suppl. Table S2: primary and secondary antibodies used for Western blotting.
