# Supplementary material for: A Positive Feed Forward Loop between Wnt/β-Catenin and NOX4 Promotes Silicon Dioxide-Induced Epithelial-Mesenchymal Transition of Lung Epithelial Cells
Source: Oxid Med Cell Longev. 2020 Dec 8;2020:3404168. doi: 10.1155/2020/3404168 (PMC7744200; doi:10.1155/2020/3404168)
Supplement: Supplementary Materials — Suppl. Table S1: primary and secondary antibodies used for immunostaining. Suppl. Table S2: primary and secondary antibodies used for Western blotting. [file 3404168.f1.pdf]

Suppl. Table 1. Primary and secondary antibodies used for immunostaining

Protein	Vendor	Product no.	Type	Dilution	Figure
Wnt3a	Abcam	ab28472	Rabbit polyclonal	1:100	Fig.1 and Fig.2
Active-β-catenin	CST	#8814	Rabbit monoclonal	1:200	Fig.1 and Fig.2
α-SMA	Abcam	ab5694	Rabbit polyclonal	1:200	Fig.1 and Fig.3
Vimentin	CST	#5741S	Rabbit monoclonal	1:200	Fig.3
NOX4	Abcam	ab133303	Rabbit monoclonal	1:200	Fig.3
Alexa Fluor 488 Donkey anti-Rabbit	Jackson Immuno	109010		1:500	

Suppl. Table 2. Primary and secondary antibodies used for Western blotting

Protein	Vendor	Product no.	Type	Dilution	Figure
Wnt3a	Abcam	ab28472	Rabbit polyclonal	1:1000	Fig.1, 2, 4, 5 and 6
Active-β-catenin	CST	#8814	Rabbit monoclonal	1:1000	Fig.1,2, 4-6
Axin2	Proteintech	20540-1-AP	Rabbit Polyclonal	1:1000	Fig.1, 6
DKK1	Proteintech	21112-1-AP	Rabbit Polyclonal	1:1000	Fig.4-6
GSK-3β	Proteintech	22104-1-AP	Rabbit Polyclonal	1:1000	Fig.6A
MMP2	Proteintech	10373-2-AP	Rabbit Polyclonal	1:1000	Fig.4A
E-cadherin	Proteintech	20874-1-AP	Rabbit Polyclonal	1:1000	Fig.3-6
α-SMA	Abcam	ab5694	Rabbit polyclonal	1:1000	Fig.1, 3-6
Vimentin	CST	#5741S	Rabbit monoclonal	1:1000	Fig.1, 3-6
NOX1	BOSTER	BA3720	Rabbit Polyclonal	1:1000	Fig.3
NOX3	Proteintech	20065-1-AP	Rabbit Polyclonal	1:1000	Fig.3
NOX4	Abcam	ab133303	Rabbit monoclonal	1:1000	Fig.3, 5
NOX4	NOVUS	NB110-58849	Rabbit Polyclonal	1:500	Fig.1, 6
NOX5	BOSTER	BA2819	Rabbit Polyclonal	1:1000	Fig.3
Histone H3	Millipore	06-755	Rabbit Polyclonal	1:1000	Fig.6

Nrf2	Proteintech	16396-1-A	Rabbit Polyclonal	1:1000	Fig.6
HO-1	Proteintech	27282-1-AP	Rabbit Polyclonal	1:1000	Fig.6
Bcl-2	SANTA CRUZ	sc-783	Rabbit Polyclonal	1:1000	Fig.6
Bcl-xl	Proteintech	26967-1-AP	Rabbit Polyclonal	1:1000	Fig.6
caspase3	Proteintech	19677-1-AP	Rabbit Polyclonal	1:1000	Fig.6
P53	SANTA CRUZ	sc-1312	Goat polyclonal	1:1000	Fig.6
CyclinD1	Proteintech	60186-1-Ig	Mouse Monoclonal	1:1000	Fig.6
GAPDH	Bioss	BS-2188R	Rabbit Polyclonal	1:2000	Fig.1-6
β-actin	Proteintech	60008-1-Ig	Mouse Monoclonal	1:2000	
Goat-anti-Rabbit	ZSGB-BIO	ZB-2301		1:5000	
Goat-anti-Mouse	ZSGB-BIO	ZB-2305		1:5000	
Donkey-anti-goat	Jackson Imm	109291		1:5000	
